# A tale of three morphs: nectar, reproductive compatibility, and morph abundance explain reproductive success within a polymorphic ginger from Western Ghats, India

**DOI:** 10.1093/aobpla/plag029

**Published:** 2026-06-16

**Authors:** Saket Shrotri, Vinita Gowda

**Affiliations:** Tropical Ecology and Evolution (TrEE) Lab, Department of Biological Sciences, Indian Institute of Science Education and Research (IISER) Bhopal, Room 303, Academic Building 3, Bhopal, Madhya Pradesh 462066, India; Faculty of Science, Department of Biology, University of Southern Denmark, Campusvej 55, Odense 5230, Denmark; Tropical Ecology and Evolution (TrEE) Lab, Department of Biological Sciences, Indian Institute of Science Education and Research (IISER) Bhopal, Room 303, Academic Building 3, Bhopal, Madhya Pradesh 462066, India

**Keywords:** *Curcuma caulina*, bract colour polymorphism, hawkmoth pollination, laterite plateaus, path analysis, reproductive compatibility, reproductive fitness, self-incompatibility

## Abstract

Although floral colour polymorphism is common in angiosperms, the functional nature of polymorphic traits within a species remains poorly understood. Colour morphs may differ in traits other than colour, including nectar reward, pollinator visitation, and reproductive compatibility. We studied bract colour polymorphism in a nocturnal ginger, *Curcuma caulina*, to ask: (i) Do the nectar rewards, hawkmoth visitations, and reproductive compatibility differ among morphs? (ii) Do the morphs differ in their female reproductive success, and does this correlate with their observed abundance? We measured floral and vegetative traits in three dominant morphs and compared nectar rewards, nocturnal pollinator visitation rates, intra- and inter-morph compatibility, and natural fruit and seed production. The morphs did not differ in morphology but differed in their bract colour and within-population abundance. The common green–red morph has low-energy nectar, low pollinator visitation rates, and is self-incompatible, but has the highest reproductive success. The rare red–white morph, despite high-energy nectar, higher pollinator visitation rates, and a leaky self-compatibility, shows lower female reproductive success. Thus, the morphs have contrasting nectar traits, reproductive compatibility, and reproductive success. Path analyses indicated that nectar reward, pollinator visitation, reproductive compatibility, and abundance jointly explained variation in female reproductive outcomes, although their effects differed among morphs. These results show that bract colour morphs of *C. caulina* differ in multiple ecological and reproductive traits and provide a foundation for future studies testing male fitness, seedling survival, clonal structure, and abiotic selection.

## Teaser

Bract-color polymorphism of Curcuma caulina, a wild ginger from India’s Western Ghats, differs in more than its color. Among the three color morphs dominant in the population, variable traits were also identified in their nectar reward, hawkmoth visitation, reproductive compatibility, and seed production. The rare red-white morph received the most visits but produced the fewest seeds, whereas the common green-red morph showed the highest seed production. Results from this study show that plant color polymorphism cannot be explained by pollinator attraction alone. Instead, the reproductive success of these color morphs depends on a combination of ecological and reproductive traits. This study characterises striking plant polymorphisms in a wild ginger species and provides an ecological framework for future studies investigating how such polymorphisms are maintained in nature.

## Introduction

Floral colour polymorphism (henceforth FCP) is the co-occurrence of two or more colour forms within a single species. The origins of FCP are usually explained by standard genetic processes such as mutation and recombination (Stebbins 1950, Kellenberger et al. 2019), and the maintenance of morphs in any population is often explained by ecological factors such as selection by pollinators. Both vertebrate and non-vertebrate pollinators are well known for their use of floral colour as a cue to locate and choose flowers resulting in pollinator-mediated selection shaping the success and decline of morphs within a population (Rausher 2008, Schiestl and Johnson 2013, de Jager and Ellis 2014, Milano et al. 2016, Bigio et al. 2017, Dyer et al. 2021). However, there is mounting evidence that shows that floral colour morphs also vary in other traits such as morphological, physiological, reproductive, and ecological traits, which can ultimately affect the morph’s fitness (Narbona et al. 2018, Sapir et al. 2021).

In some plants with FCP, pollinator preference for a morph has been empirically shown to be weak or absent (Waser and Price 1981, Schemske and Bierzychudek 2007, Tang and Huang 2010, Keasar et al. 2016, Hassa et al. 2020), and in several studies the target of selection within a population polymorphic for its floral and reproductive traits is not their floral colour (Fry and Rausher 1997, Subramaniam and Rausher 2000, Warren and Mackenzie 2001, Dick et al. 2011, Vaidya et al. 2018, Dafni et al. 2020). These studies also show that traits such as nectar rewards, flower size and number, pollen and ovule attributes, physiological tolerances, and mating-system differences alter reproductive outcomes independently of pollinator colour choice and may play a greater role in the maintenance of the floral colour morphs (Wolfe 1993, Gómez 2000, Lau and Galloway 2004, Mu et al. 2011, Arista et al. 2013, Carlson and Holsinger 2013, Ortiz et al. 2015, Koski and Galloway 2020). For example, seed viability differences linked to colour morphs have been reported in *Boechera stricta* (Vaidya et al. 2018), while abiotic tolerance associated with colour has been implicated in species such as *Parrya nudicaulis* and *Anemone coronaria* (Dick et al. 2011, Dafni et al. 2020).

Variable morph abundances and population shifts in morph frequencies have been documented in clonal plants that show FCP (Elam and Linhart 1988, Lau and Galloway 2004, Caruso et al. 2010, Berardi et al. 2016, Jacquemyn and Brys 2020, Koski et al. 2020). However, only a few studies have examined whether colour morphs differ in reproductive compatibility, pollinator interactions, and reproductive success. Therefore, while colour morphs can often be assigned visually, interpreting their ecological significance is more difficult when the colour covaries with other floral, vegetative, reward, or compatibility traits and thus requires a complex multivariate approach (Milano et al. 2016, Twyford et al. 2018, Roguz et al. 2020). To identify characteristics of a species with FCP, we propose that it is critical to first characterize the observed morphs within the species. This includes documenting visual features such as colour, as well as other vegetative morphological traits, floral rewards, ecological interactions such as pollinator visitation, and reproductive compatibility (autogamy, self-compatibility, cross-compatibility). This kind of multidimensional empirical characterization of the morphs will allow quantification of the reproductive differences among the morphs, which may eventually lead to identification of mechanisms that maintain polymorphism in a species (Wolfe and Sellers 1997).

Here, we use comparative methods to explore characteristics of the different morphs within a polymorphic, nocturnal ginger, *Curcuma caulina*, from the family Zingiberaceae. All gingers are perennials due to their rhizomatic habit, and polymorphism is common in gingers. Several genera show pronounced intraspecific variation in flower and bract colours, and within the Asian tropics different types of floral polymorphisms (stylar, bract colour, and floral colour polymorphisms) have been reported in genera such as *Alpinia*, *Amomum*, *Curcuma*, *Hedychium*, *Globba*, and *Roscoea* (Poulsen and Lock 1997, Harris et al. 2000, Leong-Skornicková et al. 2007, Mood et al. 2013, Yadav and Gowda 2024, Yadav 2025). In some taxa, floral polymorphism has also been linked to pollinator shifts and speciation dynamics (Takano et al. 2009, Paudel et al. 2019), but detailed, multi-trait descriptions and investigations linking trait variability among morphs to their reproductive success are rare. Thus, gingers present a novel, unexplored system to evaluate the role and maintenance of floral polymorphism in perennial, rhizomatous species.

**Figure 1:**
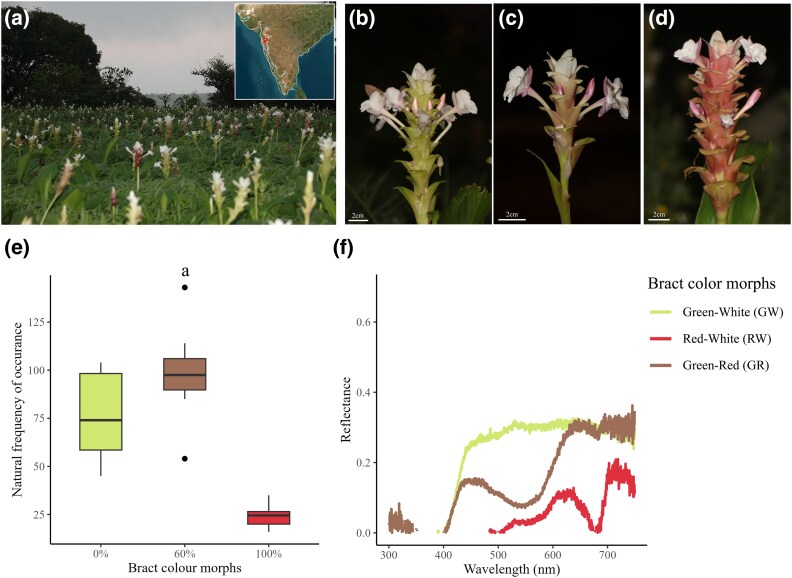
Study population, bract colour morphs, natural frequency, and spectral reflectance of *Curcuma caulina*. (a) Mass flowering of *C. caulina* at Kaas plateau, showing the sympatric occurrence of bract colour morphs in the species’ native lateritic plateau habitat. The inset map shows the geographical location of the study site in peninsular India; the Western Ghats boundary is shown. (b–d) Representative inflorescences of the three bract colour morphs: (b) green–white morph (GW; 0% red on the first lateral bract), (c) green–red morph (GR; 60% red), and (d) red–white morph (RW; 100% red). Scale bars = 2 cm. (e) Natural frequency of occurrence of the three bract colour morphs recorded from field plots. Different letters above boxplots indicate statistically significant differences among morphs. (f) Spectral reflectance profiles of the three bract colour morphs measured from the first lateral bract.

In this study, we present a comprehensive characterization of bract colour polymorphism in *C. caulina*, a ginger endemic to the laterite plateaus of the Northern Western Ghats (Fig. 1a). In our preliminary observations, we had noted that morphs showed variable abundances within a population, with three dominant and two rare morphs. Due to the absence of any prior ecological studies on *C. caulina*, we focused on three complementary sets of traits to characterize intraspecific morph variations: morphology (bract and floral display), pollination ecology (pollinator visitation), and reproductive compatibility (autogamy, cross-compatibility, and seed set). These axes capture both pre-pollination (pollinator attraction and pollen receipt) and post-pollination (fertilization and compatibility) processes and can be quantified in the field and link phenotype to reproductive success.

Since nectar reward is a primary attractant for nectar-feeding pollinators, using field experiments, we tested if morphs differed in their nectar rewards and if higher nectar reward in a morph was accompanied by higher pollinator visitation rates, higher pollen transfer, and increased fruit set and seed set. Since we had recorded differences in morph abundances within our study site, we predicted that the most common morph may show the presence of the highest nectar rewards, visitation rates, and fruit set and seed sets, as its abundance may be a reflection of its reproductive advantage. On a similar note, we also expected that rare morphs would have lower nectar reward, lower pollinator visitation rates, and lower reproductive success. Based on these expectations, we addressed the following questions:

- Do nectar rewards, reproductive compatibility, and pollinator visitations differ among the colour morphs?
- Do pollinator and plant reproductive traits explain reproductive success, and do these correlate with the observed morph abundance?

## Materials and methods

### Study species and study site

*Curcuma caulina* (Zingiberaceae) is a rhizomatous, mass-flowering (Fig. 1a), perennial herb that flowers between July and October. The plant is 30–50 cm tall with a single shoot bearing a terminal inflorescence, which consists of 10–18 fertile ‘lateral bracts’ that bear flowers (Supplementary Figure S1). Flowers are born in cincinni (1–3 flowers per cincinnus), are nocturnal (anthesis at 18:00), and last 12–14 hours only. The stigma is funnel-shaped and projects beyond the anther, and the fruits mature within the bracts. *Curcuma caulina* is endemic to the northern Western Ghats of India, and this study was carried out on the Kaas plateau, Maharashtra (elevation 1225 m, Fig. 1a) between 2019 and 2022. The Kaas plateau is an elevated ferricrete outcrop (red laterite crust) and a UNESCO Natural World Heritage site recognized for its floral diversity and high endemicity (Kulkarni et al. 2023, Shrotri et al. 2025). *Curcuma caulina* is a rhizomatous and perennial species, and the vegetative structure can re-emerge across flowering seasons. However, the genet lifespan and the number of reproductive seasons per genet are not known for this species. We therefore interpret our results at the level of observed flowering shoots and do not infer long-term population demography.

### Bract colour polymorphism and variation in floral morphology and nectar rewards

The colour of the lateral bracts of *C. caulina* individuals varied within the population from predominantly green to predominantly red coloured inflorescence (Supplementary Figure S2). To determine the frequency of bract colour morphs, we identified and counted all morphs within 10 randomly placed plots, each measuring 10 × 4 m^2^. Variations in the colour of the lateral bracts were first identified based on the extent of the reddish-pink colouration (0%–100%) on the first or basal lateral bract. This colour difference was visually identified and also quantified using a hand-held spectrophotometer (Ocean Optics).

To morphologically characterize the morphs, we measured a total of 22 vegetative and floral characters using digital vernier callipers and rulers (*n* = 30 per morph; a character list provided in the Supplementary Table S1). To assess morphological variation among the morphs, we ran a non-metric multidimensional scaling (nMDS) analysis with Bray–Curtis dissimilarity using the VEGAN package (Dixon 2003) in R (4.3.0; R Core Team 2023). We used the first three dimensions to construct three-dimensional (3D) plots, which were used to visualize both intra-morph and inter-morph variations. The statistical difference among the overlapping clusters (morphs) was measured using the ANOSIM test, where the Bray–Curtis dissimilarity matrix with 1000 permutations was used (VEGAN package; Dixon 2003).

We measured nectar volume and concentration in 106 bagged flowers (32–38 unopened buds per morph) between 18:00 and 18:30 hours by destructive sampling. Mature unopened buds were bagged at 15:00 hours before anthesis. Bags were retained until nectar sampling between 18:00 and 18:30 hours. The nectar volume (μl), concentration (% sucrose), and nectar energy (cal) were compared among the three morphs. Nectar volume was measured using calibrated glass microcapillary tubes (Drummond Scientific Company), and the sucrose concentration in nectar was determined using a hand-held refractometer (ERMA BRIX 0∼55%). The brix sucrose % value was then converted into sugar (mg) per 1 ml of nectar using the table given in the CRC Handbook of Chemistry and Physics (Lide 2004) and as outlined by Bolten et al. (1979). This value was used to calculate the amount of sugar in a given volume of nectar, and it was converted into calories (1 mg of sucrose = 4 calories as given in Heinrich 1975). Differences between the morphs were tested using the Kruskal–Wallis test followed by Dunn’s test as a *post hoc*; the significance was set at *P* < .05; analysis was carried out using Base R (4.3.0; R Core Team 2023).

### Natural pollinator visitations

To determine if pollinators exhibit differential visitation towards a particular morph, pollinator visits were monitored by night vision CCTV cameras (Ae Zone CCTV IR day/night vision USB camera) between 18:00 and 06:00 hours. These observations were carried out in 18 randomly selected observation plots on the plateau, where each plot consisted of three to six individuals. All morphs were observed for a total of 648 hours (i.e. 216 hours per morph). Cameras were positioned approximately 1–1.5 m away from the focal plants such that all inflorescences were visible in their entirety in the field of view. The recordings were manually scored, where the total number of visits by each pollinator and the total number of flowers present in the plot were recorded. We calculated the pollinator visitation rate as the total number of visits per flower per hour for each morph. The visitation rates were summed for every hour between 18:00 and 06:00 hours in order to record the temporal shift in pollinator visitation rate.

Natural pollinator visitation rates (number of visits per flower per hour) were compared among the morphs on a temporal scale using the circular statistical analysis in ORIANA (v. 4.02 Kovach Computing Services; Kovach 2011). Temporal differences in visitation rates between the morphs were tested using the Mardia–Watson Wheeler test (or uniform score test; Zar 1999). To test for inter-morph differences in pollinator visitation rates, we used the peak visitation rates from 19:00 to 22:00 hours, which accounted for ∼85% of all pollinator visits. We used the Kruskal–Wallis test followed by Dunn’s test as a *post hoc* test at *P* < .05. All statistical analyses were carried out using Base R (4.3.0; R Core Team 2023).

### Reproductive compatibility and natural fruit set

To comprehensively evaluate the self-compatibility and cross-compatibility rates in the three floral colour morphs, we carried out a total of 15 hand-pollination treatments. Since we identified three bract colour morphs in this population (refer to results for within population morph variability), reproductive compatibility was tested for these three morphs, where one morph was rare, and two were common. Each morph was treated separately, and the pollination treatments included three autogamy (autonomous development of fruits without manual pollen transfer), three self-pollination (manual pollen transfer), and nine cross-pollinations that included three intra-morph cross-pollinations and six inter-morph cross-pollinations where the identity of a morph as the male or female parent was recorded.

Autogamy treatment included unmanipulated, bagged flowers that were checked for fruit set after 4 weeks. For cross-pollination treatments, flowers were emasculated, and mixed pollen grains from 10 to 15 individuals were manually transferred on marked flowers. Flowers were observed for fruit set in ∼4 weeks, and female reproductive success (as percentage) was measured for each treatment using the number of fruits per treatment as well as the number of seeds per fruit (seed count). We statistically tested the female reproductive success rates between self- and cross-pollination treatments for each morph and compared male versus female reproductive success for the morphs using Fisher's exact tests. Comparative analyses were also carried out for the seed counts from self- and cross-pollination treatments using the Kruskal–Wallis test followed by Dunn’s *post hoc* test with significance set at *P* < .05.

We quantified the female reproductive success of natural pollination events within the study population at the end of the flowering season in mid-October by measuring fruit set in ≥500 individuals per morph and seed counts in ≥50 fruits per morph. For each inflorescence, we estimated the total number of flowers per inflorescence by multiplying the number of lateral bracts by the number of flowers estimated in a cincinnus. Fruit set per inflorescence was then calculated as the ratio of the number of mature fruits to the total number of flowers estimated for a particular inflorescence. We used the Kruskal–Wallis test to compare both natural fruit set and natural seed count among morphs. Dunn’s *post hoc* test was used with a significance set at *P* < .05.

### Path analysis to examine morph-specific trait variation and reproductive success

We used path analysis to examine the direct and indirect effects of morph abundance on natural fitness, as described by Mitchell (1993). Path analysis was tested across the entire Kaas plateau population of *C. caulina* (population-level model) and for each morph (morph-level model) to assess the net effect and important selection regimes within the population and within each morph. The path analysis consisted of four major paths starting from the first variable, namely relative morph abundance, to the final variable, reproductive fitness. Since morph abundance and nectar reward are known to affect pollinator visitation rates, the first path (*a*) explains the role of abundance on pollinator visitation rate, and the second path (*b*) explains the role of nectar reward on pollinator visitation rate. We next defined the product of the fruit set and seed set in each morph as reproductive fitness. Since the success of pollinator visitation rates can be measured in terms of reproductive outcomes, the third path (*c*) explains the effect of pollinator visitation rates on reproductive fitness, and the fourth path (*d*) explains reproductive fitness due to the type of reproductive compatibility (self or cross). The fourth path is critical in our study, as we noted that the outcome of the type of pollination event (self or cross) depends on reproductive compatibility, which was found to differ among the three morphs. Finally, using path *e*, we tested the putative role of morph abundance on reproductive fitness.

Path analysis was performed in R (4.3.0; R Core Team 2023) using the *lavaan* package (Rosseel 2012), and path estimates were computed via 10 000 bootstrap iterations. Estimates of model fit were computed using the chi-squared goodness-of-fit statistic, the Comparative Fit Index (CFI), and the Tucker–Lewis Index (TLI). A non-significant chi-squared value indicates that a model is not significantly different from the observed correlations in the data and therefore provides a good fit, whereas CFI and TLI indices above 0.9 are considered a good fit (Mitchell 2001). Finally, we tested the hypothetical relationship (path *f*) between reproductive fitness and relative abundance using a regression model, which was not part of the main path analysis.

## Results

### Floral (bract) colour polymorphism and morphological variability

We initially recognized six bract colour variants based on the approximate proportion of red pigmentation on the basal lateral bract: 0%, 15%, 30%, 45%, 60%, and 100% red (Supplementary Figure S2a). However, using Ford’s criteria for polymorphic forms (Ford 1945), we chose the three most common variants (0% red, 60% red, and 100% red; henceforth referred to as ‘morphs’) in this study. These three morphs were named based on the colouration of their lateral and coma bracts as follows: the 0% variant is green–white (GW) morph (Fig. 1b), the 60% variant is green–red (GR) morph (Fig. 1c), and the 100% variant is red–white (RW) morph (Fig. 1d). Since the 45% morphs were very rare (Supplementary Figure S2b) and difficult to distinguish from the 60% morph identified as the GR morph, the 45% morph is not considered as a separate morph and is treated as the GR morph. The abundances of the three morphs were unequal (Fig. 1e and Supplementary Figure S2), with GR morphs being the most common (∼44% occurrence), followed by GW (∼29%) and RW (13%). The remaining two bract colour variants (15% and 30%) were extremely rare, each constituting <5% of the population and therefore they were not used in this study (Supplementary Figure S2). We noted that the three morphs show distinct spectroscopic reflectance and can also be identified using hand-held spectrophotometry (Fig. 1f). We also noted that bract colour morphs were true to the rhizome (or the individual) and did not alter when plants were transplanted from the field to the controlled environment of a shade house.

The abundance of the three morphs differed within the population (Fig. 1e). The GR morph (98.6 ± 7.2 individuals; ∼44%) and GW morph (75.7 ± 7.2 individuals; ∼29%) were the most common morphs; their frequencies were not significantly different from each other (Kruskal–Wallis χ^2^ = 0.692, df = 1, *P* = .2446). The rarest of the three morphs was the RW morph with 24.2 ± 2.0 individuals (∼13%) in the population, and the RW morph had significantly fewer individuals when compared to both GR and GW (RW vs. GR, Kruskal–Wallis χ^2^ = 2.427, df = 1, *P* = .0076 and RW vs. GW, Kruskal–Wallis χ^2^ = 1.736, df = 1, *P* = .0413, respectively). All other bract colour variants totalled to roughly 10% in the population and thus were considered rare. The nMDS analysis of the 22 floral and vegetative morphometric characters (Supplementary Table S1 and Supplementary Figure S3) failed to form distinct clusters separating the morphs by their morphological traits. Furthermore, the Analysis of Similarities (ANOSIM) test (*P* = .4835) and the associated ANOSIM *R*-value (ANOSIM *R* = −0.0038) also indicated no statistical significance in the differentiation of these clusters for each morph. Since we detected polymorphism in bract colours and not floral colours, henceforth, the term colour polymorphism refers to only bract colour polymorphism in *C. caulina*.

### Variability in nectar traits and hawkmoth visitation rates

**Figure 2:**
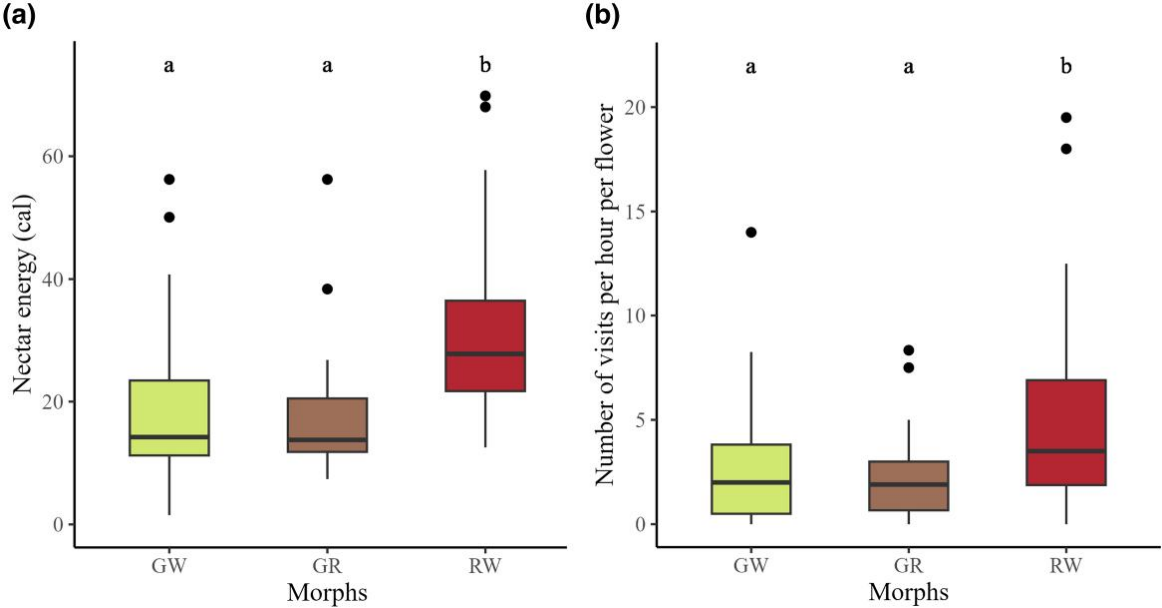
Nectar energy and natural pollinator visitation rates for three bract colour morphs of *Curcuma caulina*. Boxplots for (a) nectar energy per flower and (b) natural pollinator visitation rate, measured as the number of visits per flower per hour, for the green–white (GW), green–red (GR), and red–white (RW) morphs. Different letters above boxplots indicate statistically significant difference among morphs.

Nectar is the primary pollinator reward in *C. caulina*, and there was no significant difference between the total nectar rewards (assessed in calories as mean ± SE) of the two common morphs GR (16.74 ± 1.47) and GW (18.04 ± 2.2). However, the total nectar reward was highest for the rare RW morph (31.31 ± 2.25; Fig. 2a) due to both its higher nectar volume and concentration (Supplementary Table S2; Kruskal–Wallis χ^2^ = 33.53, df = 2, *P* < .0001). Flowers of *C. caulina* were visited by two species of hawkmoths with long probosces: *Agrius convolvuli* (∼12.4 cm) and *Hippotion rafflesii* (∼4.6 cm; Sphingidae; Supplementary Figure S4). During foraging, the moths insert their proboscis into the floral tube, hover over the flower, and the sticky pollen is carried on the proboscis of *A. convolvuli* (Supplementary Figure S4) or on the head in the case of *H. rafflesii*. We did not observe the curling of proboscises during foraging, as noted by Smith et al. (2022). The average time spent by each pollinator per flower was ∼1.5 s for *A. convolvuli* and ∼ .5 s for *H. rafflesii*, and the rupture in anther theca could be used as a visual identifying feature for flowers visited by hawkmoths.

The circular statistical analysis of visitation rates across the anthesis time showed no temporal differences in pollinator visitations among the three morphs (Rayleigh test; *P* < .0001; Supplementary Figure S5). The mean pollinator visitation time, denoted by the circular mean vector µ, was noted to be between 21:00 and 22:00 for all morphs (Supplementary Figure S5). All three morphs showed the highest hawkmoth activity during the first few hours of anthesis, between 19:00 and 23:00 hours, which gradually decreased until there was no activity by 06:00 hours the following morning (Supplementary Figure S5). Pollinator visitation rates of the three morphs during the peak visitation hours were statistically different from each other with RW morph showing the highest visitation rate (mean number of visits per flower per hour ± SE; 5.14 ± 0.96), followed by the GW morph (2.8 ± 0.59), and GR morph (2.19 ± 0.3; Fig. 2b; Kruskal–Wallis chi-square = 6.52; df = 2; *P* = .03839; Supplementary Table S3).

From video recordings of pollinator visitations, we also observed that hawkmoths revisited the same flowers of the RW morph (∼10–12 times), once they discovered this rare but nectar-rich inflorescence. In contrast, the two common morphs GR and GW with low nectar rewards received lower visitation rates, and pollinators rarely revisited the flowers. Thus, within-inflorescence visits were higher in the rare RW morph compared to the common GR and GW morphs.

### Variability in the reproductive compatibility and natural fitness among the morphs

A total of 471 hand pollinations were carried out (with *n* ≥ 30 per treatment) where the three intra-morph cross-pollination treatments were: GW × , GR × , and RW × (male × female morph) and will be interchangeably referred to as ‘cross-pollination’ treatments. Inter-morph cross-pollination treatments were categorized into three pairs to investigate the effect of male and female parents in each treatment. These crosses are (male × female) identified as pairs a: GW × GR and GR × GW, b: GW × RW and RW × GW, and c: GR × RW and RW × GR. Out of 471 hand-pollination treatments, only 108 treatments set fruits (Supplementary Tables S4 and S5). None of the autogamous treatments set fruits. Selfing treatments failed to set fruits exclusively in the GR morph, whereas the GW and RW morphs demonstrated leaky self-compatibility with success rates of 6.67% and 9.68%, respectively. Fruit-set success in selfed treatments was 5.8%, which was significantly lower (χ^2^ = 31.299, *P* < .0001; Supplementary Table S4) when compared with fruit-set success in the intra-morph cross (48.38%) and inter-morph cross (58.4%). The GR morph demonstrated the highest fruit set in the intra-morph outcrossed treatment with a success rate of 46.67%, followed by the RW morph at 27.03% and the GW morph at 18.75% (Supplementary Table S4).

In the inter-morph crosses, we noted donor-specific differences in the GW and RW morphs. The donor specificity was related to whether a morph was used as a male parent or a female parent in the inter-morph cross-pollination treatments. In crosses where GW morph was the male parent (i.e. pollen donor), success rates were significantly lower (pair a: GW × GR, 24.24% and in pair b: GW × RW, 25%) when compared to crosses where it was used as a female parent, that is the pollen recipient (in pair a: GR × GW, 43.33% and in pair b: RW × GW, 44.44%, Fisher’s exact test *P* = .027, odd ratios = 0.419; Supplementary Tables S4 and S5). Whereas when RW acted as the male parent (pollen donor) in inter-morph crosses, fruit set success was higher (pair b: RW × GW = 44.44%; pair c: RW × GR = 46.67%) compared to when it served as the female parent (pair b: GW × RW = 25%; pair c: GR × RW = 37.84%); however, Fisher’s exact test did not yield a significant difference (*P* = .1129, odd ratios = 1.8; Supplementary Tables S4 and S5).

**Figure 3:**
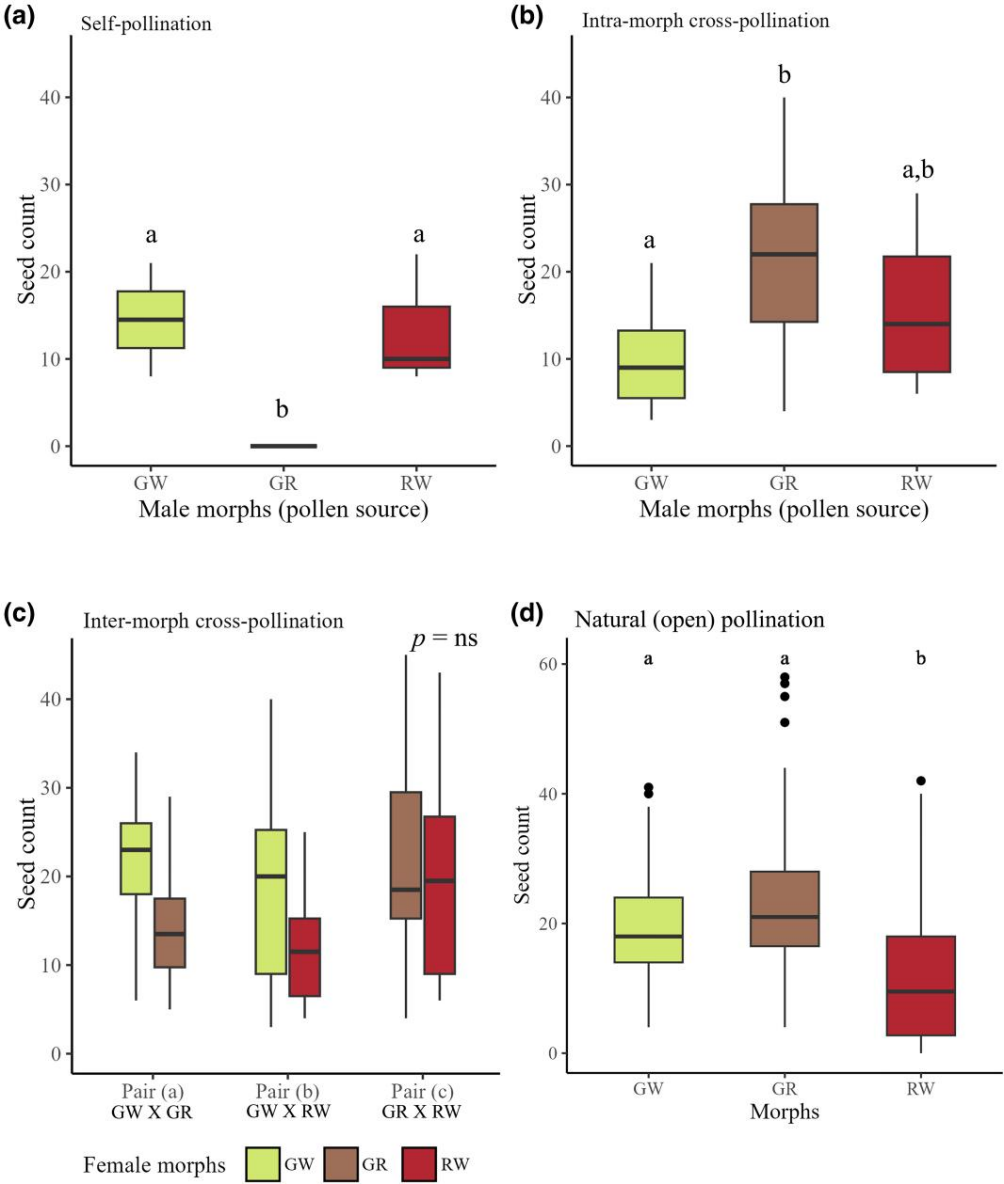
Reproductive compatibility and natural female reproductive success in the three bract colour morphs of *Curcuma caulina*. Boxplots show seed count per successful fruit following (a) manual self-pollination, (b) intra-morph cross-pollination, (c) inter-morph cross-pollination, and (d) natural open pollination. Different lowercase letters above boxplots indicate statistically significant differences among morphs; n.s. indicates no significant difference. Details of fruit-set success and statistical tests are provided in the Supporting Information.

Among all hand-pollination treatments, self-pollination treatments exhibited the lowest seed count (mean number of seeds per matured fruit ± SE; 8.63 ± 3.15), followed by intra-morph crosses (16.93 ± 1.8) and then inter-morph crosses (18.63 ± 1.2). In the self-pollination treatment, the GR morph exhibited a significantly lower seed count (zero) compared to the GW morph (14.5 ± 6.5) and RW morph (13.33 ± 4.37; Kruskal–Wallis χ^2^ = 5.3534, df = 2, *P* = .06879; Fig. 3a; Supplementary Table S6). In the intra-morph cross-pollination treatment, the seed counts varied significantly among the morphs. The GW morph exhibited the lowest seed count (10.72 ± 2.7) compared to the GR morph (20.71 ± 2.8) and the RW morph (15.7 ± 2.7; Kruskal–Wallis χ^2^ = 5.2499, df = 2, *P* = .07244; Fig. 3b; Supplementary Table S6). Seed counts in inter-morph cross-pollination treatments were not significantly different among the three pairs (Fig. 3c; Supplementary Table S6). Finally, the mean total fruit set per plant in open pollination treatments was recorded to be equal in all three morphs (Supplementary Table S6). However, the mean seed count per fruit was significantly different among the morphs (Kruskal–Wallis χ^2^ = 36.3222, df = 2, *P* < .0001) with the GR morph being the highest (23.7 ± 1.5), followed by GW (20.3 ± 1.2) and RW (11.5 ± 1.4; Fig. 3). We also noted that the seed counts recorded in the open (natural) pollination and self-pollination treatments of RW were similar (Fig. 3).

### Morph-specific trait variation and female reproductive success

**Figure 4:**
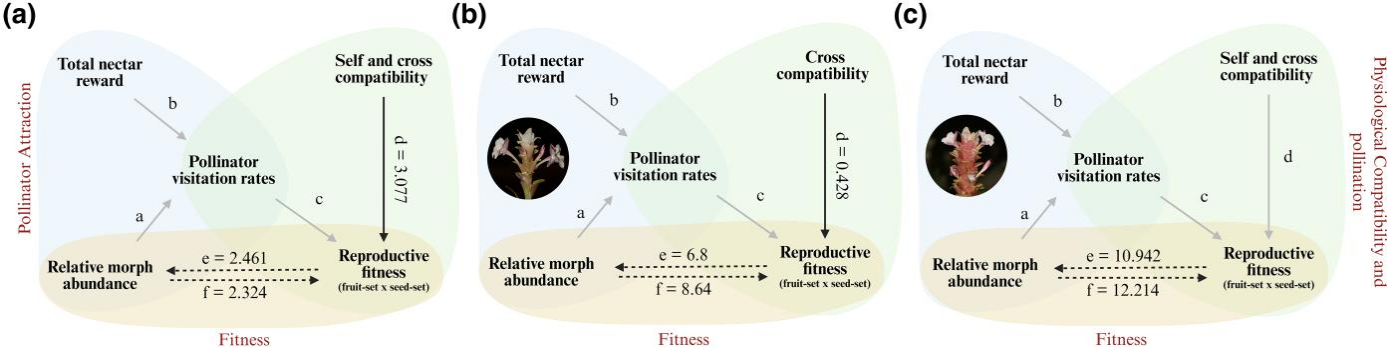
Direct and indirect associations among ecological and reproductive traits in *Curcuma caulina*. Path models are shown for (a) the species-level model, (b) the common green–red morph (GR), and (c) the rare red–white morph (RW). Arrows indicate hypothesized relationships among relative morph abundance, total nectar reward, pollinator visitation rate, reproductive compatibility, and female reproductive success, measured as fruit set × seed set. Arrows with path-estimate values indicate statistically significant paths (*P* < .05), whereas arrows without path-estimate values indicate non-significant paths. Dashed arrows indicate the hypothesized association between relative morph abundance and female reproductive success. Path labels correspond to the paths reported in Table 1, where path estimates and statistical values are provided.

In the path analyses, the five variables resulted in five direct paths (*a–e*; Fig. 4) and one indirect path representing a putative link between abundance and reproductive fitness (path *f*). The chi-squared goodness-of-fit test, CFI, and TLI scores identified a good fit for three out of the four models (*C. caulina* species model, and models for GR morph and RW morph; Supplementary Table S7). In the case of the GW morph, the model fit as estimated by TLI values was poor (TLI = −0.65), and we did not find any significant relationships between the traits as well as for the net effect. We found that both the indirect effect (path *f* in Fig. 4) and the net effect of morph abundance on reproductive fitness (path *e*) were positive and statistically significant in three (*C. caulina* species model, and models for GR morph and RW morph) of the four models (Table 1).

**Table 1:** Path estimates for all four structural equation models (species and three individual morphs).

Variables	Path ID	Population	GR morph	GW morph	RW morph
Pollination visitation rate ∼ Relative abundance	*a*	1.058	−8.15	−2.84	−3.509
Pollination visitation rate ∼ Total nectar reward	*b*	0.56	1.221	0.664	−1.327
Reproductive fitness ∼ Pollinator visitation rates	*c*	0.004	0.048	0.019	0.08
Reproductive fitness ∼ Self-compatibility	*d*	3.077*	NA	1.922	8.672
Reproductive fitness ∼ Cross-compatibility	*d*	0.092	0.428*	0.121	−0.341
Relative abundance ∼ Reproductive fitness	*e*	2.324*	8.64*	−3.21	12.241*
Reproductive fitness ∼ Relative abundance	*f*	2.461*	6.800*	−2.871	10.942*
Net total effect of relative abundance on reproductive fitness	Total	2.302*	4.814*	−3.219	12.045*

Path IDs are the same as Fig. 4 and tilde (∼) represents regression between two variables. Statistically significant interactions are marked by asterisk (*).

In the species-level model, both direct and indirect predictors were identified that affected seed production. The model indicated that morph abundance had a positive effect on nectar reward (path *a*; Fig. 4a) and a small direct positive effect on seed production (path *e*). Nectar reward showed a negative direct effect on seed production (path *g*; significant only for GR morph; Fig. 4b). Self-compatibility (hand-pollination treatments) had a positive direct effect on seed production (path *d*). After accounting for nectar and mating systems, pollinator visitations had a positive but non-significant direct effect on seed production (path *f*; Table 1). The net effect of morph abundance on reproductive fitness was positive and significant in three out of four models (*C. caulina* species model, and models for GR morph and RW morph; Fig. 4a–c, and Table 1), indicating that abundant morphs tend to have higher fitness when direct and indirect pathways are considered together (Table 1).

## Discussion

In this study, we quantified trait variation among morphs that differed in their bract colours in the perennial ginger *C. caulina*. The principal empirical findings from our study are: (i) The rare RW morph produces the largest nectar reward and receives the highest pollinator visitation, yet it attains the lowest seeds per fruit under natural pollination. (ii) The common GR morph is functionally self-incompatible and produces the highest seeds per fruit. (iii) Inter-morph crosses yield higher fruit and seed set than self-pollination or intra-morph crosses, but the magnitude of these effects differs among the morphs. (iv) Species-specific path models identified morph abundance, nectar investment and mating system to jointly predict observed female reproductive success, whereas morph-specific path models showed heterogeneous contributions of these variables. These results confirm that floral polymorphism in *C. caulina* include a few ecological traits and reproductive traits, which together are useful in explaining the observed differences in female reproductive success among the morphs.

Unequal morph abundance has been commonly reported in plants showing FCP. Narbona et al. (2018) noted that more than 80% of Mediterranean species with FCP exhibit skewed morph distributions that often fluctuate spatio-temporally due to abiotic pressures (Dormont et al. 2010, Tang et al. 2016). In *C. caulina*, we noted unequal abundance of bract colour variants, and we speculate that the three morphs may have been selected under variable abiotic selection pressures along different time periods. For example, anthocyanin-rich red bracts of RW morph may have conferred UV or drought tolerance (Berardi et al. 2016), which can confer survival advantages in the extreme environments of the rocky laterite habitats. Since the rocky lateritic plateaus of Western Ghats are also known for unique edaphic factors such as shallow-depth soil conditions (Watve 2013, Kulkarni et al. 2023, Shrotri et al. 2025), selection of different colour morphs in *C. caulina* by abiotic factors is a plausible hypothesis which can be tested in the future using targeted morph-specific transcriptomic studies.

The second morph-specific difference we report here is in the intra-morph and inter-morph reproductive compatibility. We identified two different reproductive strategies in two separate morphs of *C. caulina*: (i) improved reproductive success via strong self-incompatibility in the most common GR morph, and (ii) reproductive assurance via self-compatibility in the rare RW morph. To the best of our abilities, we are not aware of examples for the first reproductive strategy, i.e. common morph showing self-incompatibility. However, empirical evidence of rare morph showing self-compatibility in a polymorphic system has been reported in *Ipomea purpurea* (Fry and Rausher 1997, Subramaniam and Rausher 2000), *Lysimachia arvensis* (Arista et al. 2013, Ortiz et al. 2015), and now in this study on *C. caulina*. In *C. caulina* we also noted in the path analyses (path *d*) that the role of morph-specific reproductive compatibility in determining the reproductive fitness of the morphs was significant, suggesting the importance of this trait in determining differential fitness among the morphs of *C. caulina*.

Floral colour variation is widespread in Zingiberales and within the family Zingiberaceae many species exhibit striking floral and bract colour variation (Temeles et al. 2000, Leong-Skornicková et al. 2007, Gowda and Kress 2013, Maas-Van De Kamer et al. 2016, Saryan et al. 2020). Studies of the neotropical *Heliconia caribaea* and *H. bihai*, and the *trans*-Himalayan *Roscoea* spp. have suggested that specialization to specific pollinators can result in different types of plant–pollinator interactions, such that based on the pollinator visitation patterns to the different morphs we may identify one pollinator–one morph specialization (Temeles et al. 2013), or one pollinator–multi-morph specialization (Martén-Rodríguez et al. 2011, Gowda and Kress 2013, Paudel et al. 2019). In *C. caulina*, we identify the one pollinator–multi-morph specialization, where hawkmoths show differences in visitation rates to the different morphs.

Pollinator sorting and differential reproductive fitness have been shown to be shaped by morphological trait differences in tube length or flower size among morphs (Melendez-Ackerman and Campbell 1998, Meléndez-Ackerman et al. 2005; Gómez 2000, Majetic et al. 2009, Milano et al. 2016). However, in *C. caulina*, we report morph-specific differential reproductive fitness not due to trait differences in floral size and tube length or pollinator sorting, but due to pollinator visitation rate differences mediated by differences in nectar reward, type of reproductive compatibility of the morph, and morph abundance.

We also noted unique trade-offs between nectar traits and reproductive fitness among the morphs of *C. caulina*. For example, the rare RW morph shows low reproductive success despite having high-energy nectar rewards and high pollinator visitation rates, while the common GR morph shows higher reproductive success despite producing low-energy nectar rewards and lower pollinator visitation rates. This contrasts the patterns noted in the polymorphic *Ipomoea purpurea* where rare, high nectar morph directly gains fitness benefits in terms of seed set (Fry and Rausher 1997). In inter-morph crosses, RW showed a non-significant tendency towards higher fruit-set success when used as a pollen donor than when used as a pollen recipient, whereas GW showed significantly lower success as a pollen donor than as a pollen recipient. While these results indicate donor–recipient asymmetry in hand-pollination compatibility, they do not provide direct evidence of male reproductive success under natural pollination. Since higher pollinator visits can mean higher pollen export, we speculate that the rare RW morph despite lower fruit set and seed set may gain an overall higher reproductive fitness via increased pollen export (male fitness), a strategy similar to that noted in *Antirrhinum* sp. (Jones and Reithel 2001). We acknowledge that mechanisms such as differences in male reproductive fitness, seed or seedling survival, long-term environmental selection, or clonal structure can also affect morph success, which need to be explored using a demographic and genetic approach.

FCP in *C. caulina* is unique because the difference in morph abundance from common to rare is discernible in the field. In annual plants, morph abundance is often explained by frequency-dependent selection (FDS; Gigord et al. 2001, Eckhart et al. 2006, Dormont et al. 2010). But in *C. caulina*, FDS was not detected, and it is explained in detail in the next section. In our cumulative observations of the past 15 years at the Kaas plateau, inter-annual shifts in morph abundances were absent (unpublished data, personal observations by authors). Since *C. caulina* is a rhizomatous perennial, we think that teasing apart the contributions of historical selection events and recent FDS in explaining the observed extant disparities in morph abundances requires a comprehensive molecular genetic approach. However, in the absence of genetic data, based on our ecological observations, we propose that the higher reproductive success of the common GR and GW morphs despite lower nectar energy may be a result of high outcrossing rates, since the morph is self-incompatible. In contrast, the rare RW morph ensures reproductive success despite a lower discovery rate and possible pollen limitation by producing higher energy nectar and the presence of self-compatibility. Thus, we propose a multi-trait hypothesis involving an interplay between nectar reward, pollinator attraction, and reproductive compatibility (self vs. cross), which collectively may exert an influence on the reproductive success of different morphs and subsequently their population abundance.

Although FCPs are common in perennial plants, one of the challenges in understanding FCP in rhizomatous perennial plants like *C. caulina* is the detection of FDS. This is because clonality and vegetative reproduction will mask any annual or contemporary FDS. That is, the perennial nature of the rhizomes will allow historical genotypes to remain in a population as ‘genotypic vestiges’, effectively reducing the rapid frequency shifts typically driven by contemporary selective agents as reported in species with FCP (Elam and Linhart 1988, Lau and Galloway 2004, Caruso et al. 2010, Imbert et al. 2014, Barrett 2015, Berardi et al. 2016, Streinzer et al. 2019, Jacquemyn and Brys 2020, Koski et al. 2020, Roguz et al. 2020, Labin et al. 2026), in heterostylous species (Eckert and Barrett 1993, Eckert et al. 1999, Barrett 2015), and in dioecious species (Irish and Nelson 1989, Petzold et al. 2013). The importance of vegetative propagation has been noted in other gingers (Záveská et al. 2012, Skopalíková et al. 2023, Yadav 2025) and has been predicted to reduce the intensity of selection otherwise expected in sexually propagated plants (Jesson and Barrett 2002, Harder and Barrett 2006). Therefore, population models for morph-specific selection in perennial polymorphic plants, despite observed trait differences and reproductive fitness among morphs, require a more nuanced view of genetic contributions from both sexual and vegetative strategies. In the *C. caulina* system, ongoing and future studies are geared towards a molecular approach to understand the genetic structure of the population, given the expected differences in outcrossing rates between the common self-incompatible GR morph and the rare self-compatible RW morph. This will also enable estimation of outcrossing and paternity patterns and determine whether morphs differ in realized pollen-donor success.

## Conclusions

Our study provides detailed morphometric, ecological, reproductive, and distributional characterization of morphs within the polymorphic rhizomatous perennial ginger *C. caulina*, and our results are critical in highlighting: (i) morph selection and differentiation in perennial tropical rhizomatous species, and (ii) morph selection as a response to abiotic stress such as drought and heat, in the context of climate-change scenarios. We show that *C. caulina* morphs vary in their traits, and that no single trait acts as a causal factor that can explain fitness variation among the morphs. Since demographic legacies and selection regimes can only be tested using paternity analysis, population genetic tools, and knowledge about the hereditary nature of polymorphic traits, the multi-trait, ecological study presented here equips us with an empirical foundation to propose questions related to genetic diversity, dispersal, and selection regimes within this polymorphic species. Polymorphism in perennial rhizomatous species has been noted by taxonomists, and ecological and reproductive differences have been suspected. The empirical evidence of inter-morph variability presented in the polymorphic *C. caulina* supports this view, encouraging future studies to test the evolutionary consequences leading to polymorphism in a species, and if rare morphs are only transient morphs on an evolutionary time scale, representing historical survivors due to their perenniality.

## Supplementary Material

plag029_Supplementary_Data

## Data Availability

The entire dataset and R codes used for analysis in this manuscript are available at: https://doi.org/10.5281/zenodo.20630411
