# Supplementary material for: A tale of three morphs: nectar, reproductive compatibility, and morph abundance explain reproductive success within a polymorphic ginger from Western Ghats, India
Source: AoB Plants. 2026 Jun 16;18(4):plag029. doi: 10.1093/aobpla/plag029 (PMC13334228; doi:10.1093/aobpla/plag029)
Supplement: plag029_Supplementary_Data [file plag029_supplementary_data.docx]

## AoB PLANTS: Supplementary information

Title: **A tale of three morphs: Nectar, reproductive compatibility, and morph abundance explain reproductive success within a polymorphic ginger from Western Ghats, India**

Authors: **Saket Shrotri and Vinita Gowda**

**Brief legends and index:**

[**Figure S1** *Curcuma caulina* plant habit with morphological characters used in the nMDS analysis.](#_heading=h.gh5e7otfqszw) 3

[**Figure**](#_heading=h.gh5e7otfqszw) [**S2** Bract color variants of *C. caulina* and their natural frequency..](#_heading=h.ttzyrjbwrc6v) 4

[**Figure**](#_heading=h.gh5e7otfqszw) [**S3** Results of nMDS analysis.](#_heading=h.jdim30ay0865) 5

[**Figure**](#_heading=h.gh5e7otfqszw) [**S4** Hawkmoth pollinators of *C. caulina*.](#_heading=h.qblv5ebaqepl) 6

[**Figure**](#_heading=h.gh5e7otfqszw) [**S5** Circular histogram and statistics of temporal variation in pollinator visitation rates.](#_heading=h.vdltc1qlhtkh) 7

[**Table S1** Floral and vegetative morphological characters used for the nMDS analysis..](#_heading=h.wkqkf9ia456i) 8

[**Table S2** Results of within-group comparisons for nectar energy, nectar volume and nectar concentration.](#_heading=h.o7h8rhdlucjt) 10

[**Table S3** Results of within-group comparisons for peak pollinator visitation rates.](#_heading=h.svqdeiwn440j) 11

[**Table S4** Results of chi-square test for self- and cross-compatibility treatments.](#_heading=h.i08bn85jfwpp) 11

[**Table S5** Success rate and mean values for seed-count for cross- and self-compatibility treatments.](#_heading=h.2uo4a126qvu5) 12

[**Table S6** Results of within-group comparisons for seed-count for cross- and self-compatibility treatments.](#_heading=h.6unxst6l12mh) 13

**Table S7** Results of Chi-square statistics……………………………………………………… 14

### [Figure](#_heading=h.gh5e7otfqszw) S1 *Curcuma caulina* plant habit with (A) vegetative and (B) floral morphological characters used in the nMDS analysis.


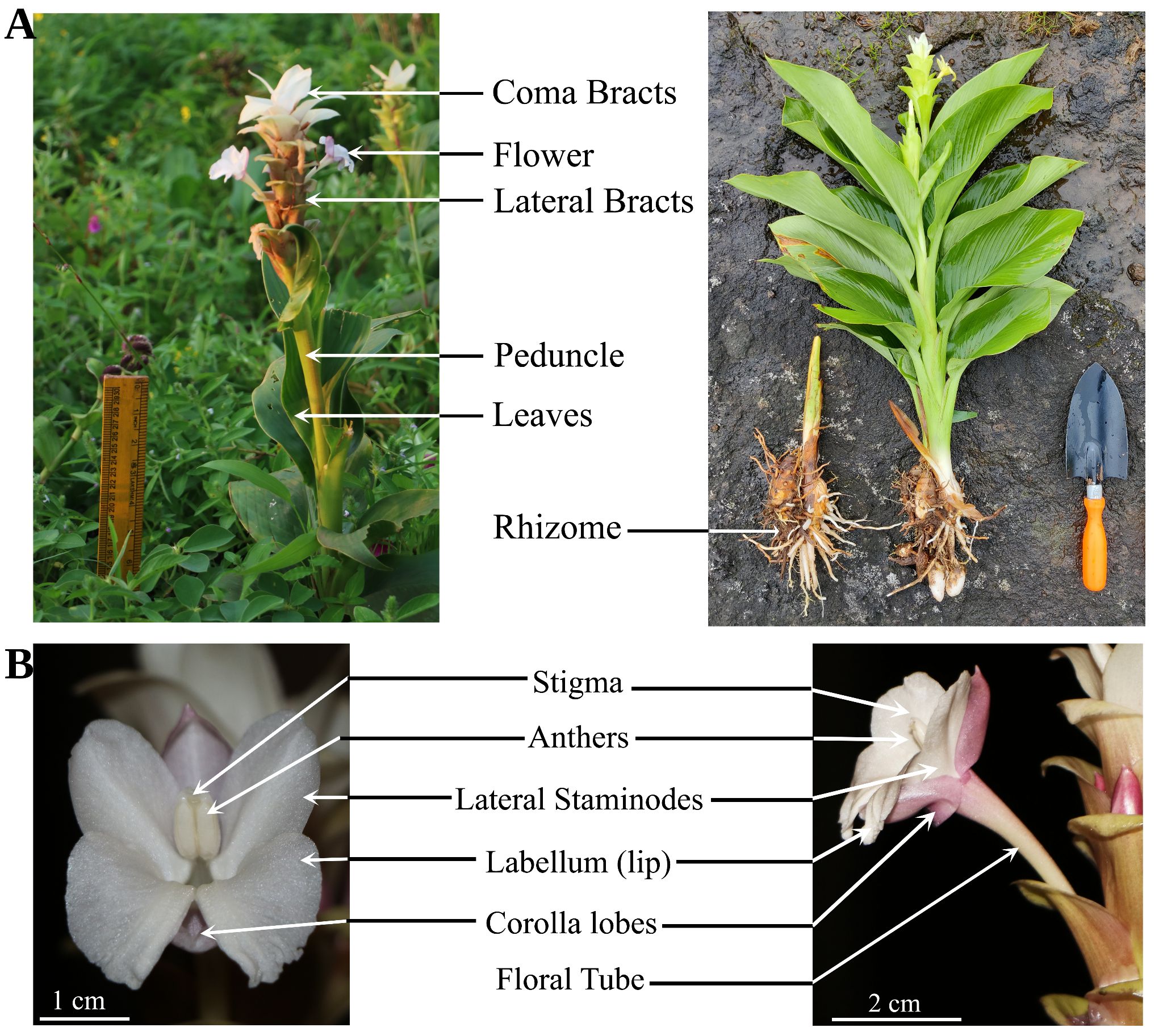


### [Figure](#_heading=h.gh5e7otfqszw) S2 Sympatric bract color variation in *C. caulina* and its natural frequency. (A) Each inflorescence represents a different individual collected from the study population. We identified six color variants based on the spread or fill of reddish-pink pigmentation on the first (or basal) lateral bract of the inflorescence. The percentage values indicate the approximate proportion of the area of this bract occupied by reddish-pink colour, ranging from 0% to 100%. Solid lines indicate the variants that were considered as three bract color morphs GW: 0%, GR: 45% and 60% and RW: 100%. (B) Mean (± s.e.) number of individuals (frequency of occurrence) of every bract color variant of *C. caulina* present in ten plots of 10m x 4m placed randomly across the study population.


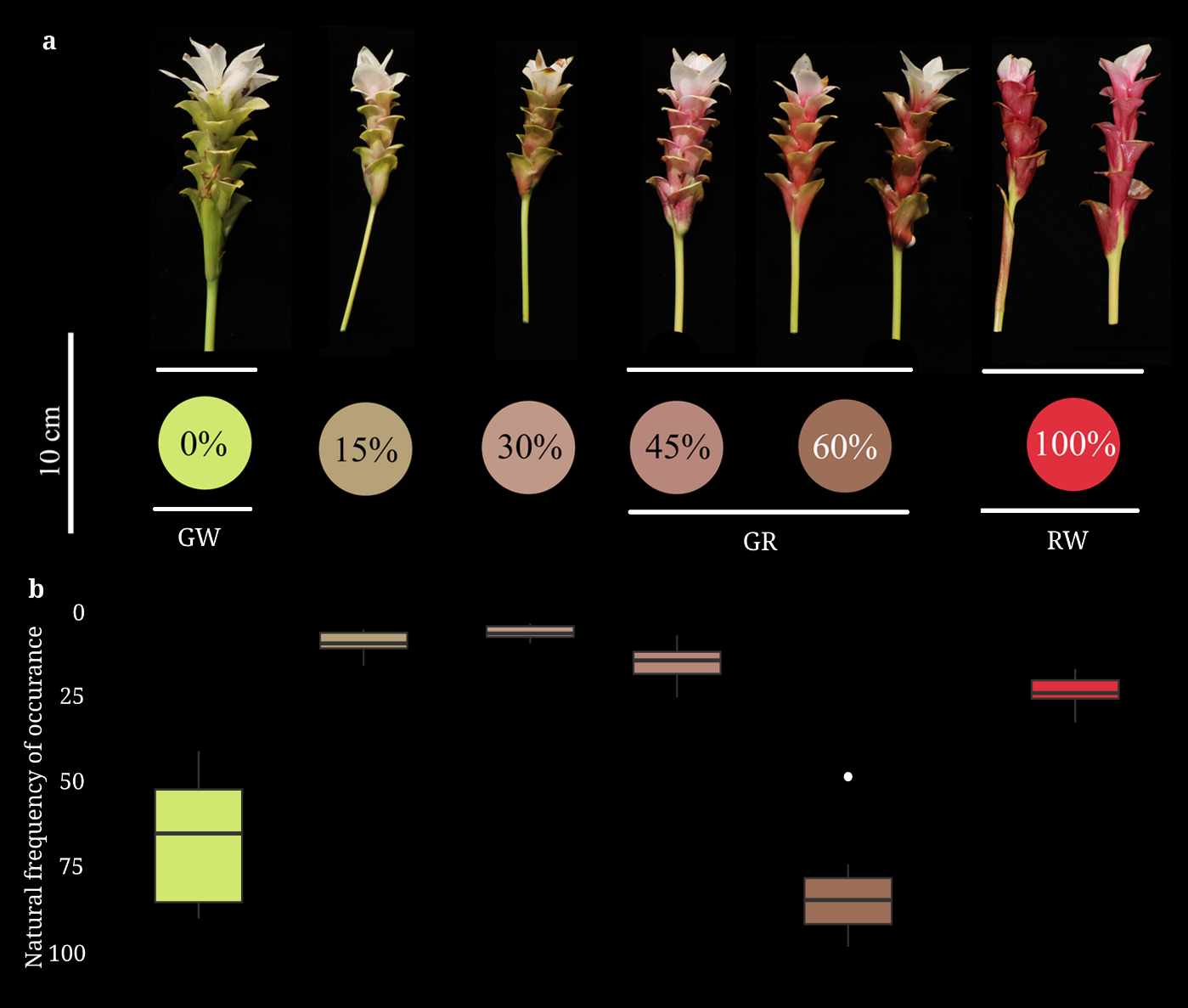


### [Figure](#_heading=h.gh5e7otfqszw) S3 Results of nMDS analysis for floral color morphs of *C. caulina* using 15 vegetative and 18 floral morphological characters. The analysis demonstrated lower stress values in a three-dimensional configuration (stress = 0.06) than in a two-dimensional configuration (stress = 0.11). The results suggest that bract color varies independently of the other morphological floral traits.


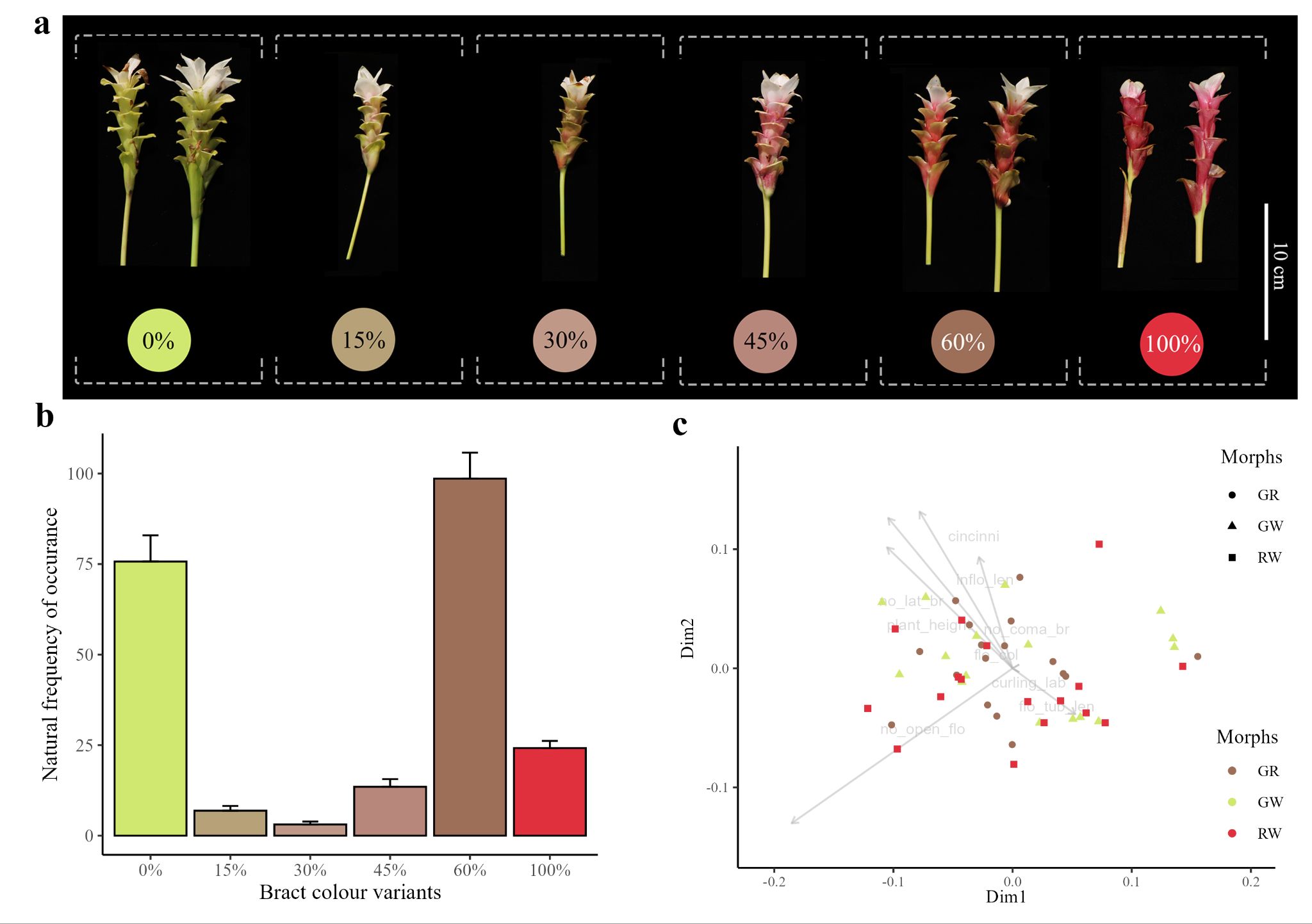


###

### [Figure](#_heading=h.gh5e7otfqszw) S4 Pollinators of *C. caulina*; a. Hawkmoth *Agrius convolvuli* visiting GR morph; b. Hawkmoth *Hippotion rafflesii* visiting RW morph, and c. pollen deposition site is the proboscis of the pollinator (shown by white arrowhead). These are the positions in which pollinators usually forage for nectar.


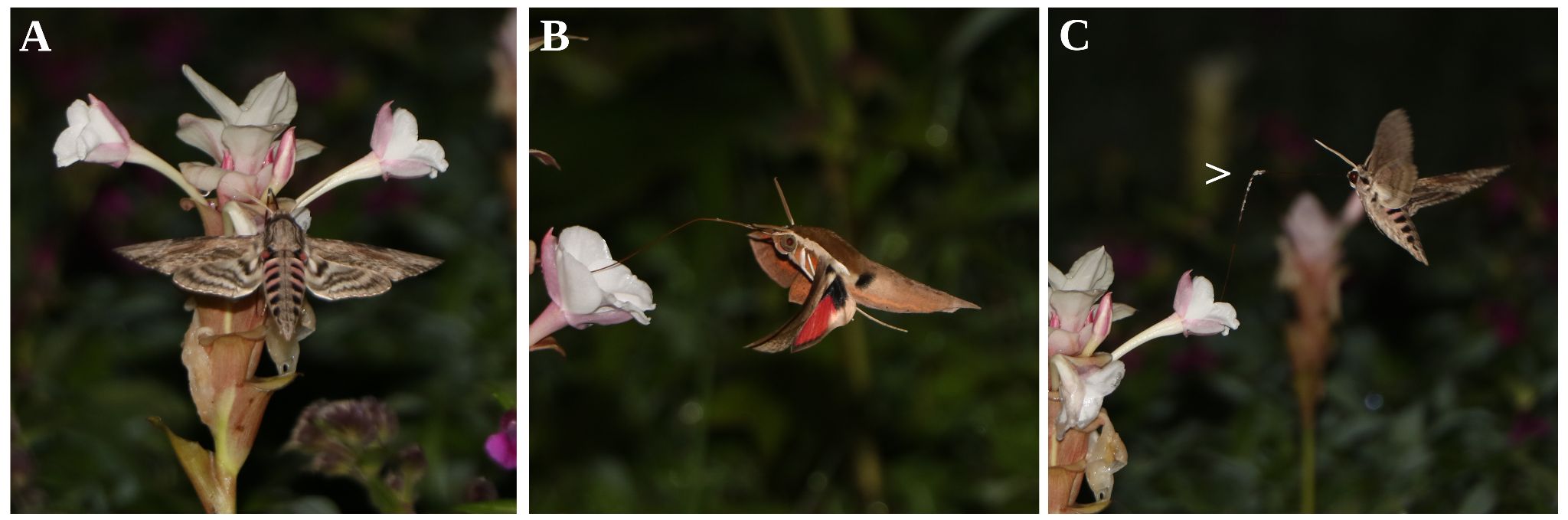


### [Figure](#_heading=h.gh5e7otfqszw) S5 Circular histogram of mean number of pollinator visits in the wild received by each morph. The 24 hours of a day are represented on the circular scale by angles with intervals of 15º. The direction of the vector (r) represents mean time and the length of this mean vector relative to the stippled line is a measure of concentration (K) of the data. The table below shows first order statistical values, results of Rayleigh test, and results of within-group comparisons (Mardia-Watson-Wheeler Test) for natural pollinator visitation rates for the three floral color morphs. The significance level was set at 0.05, with significant *p*-values indicated by an asterisk (*).


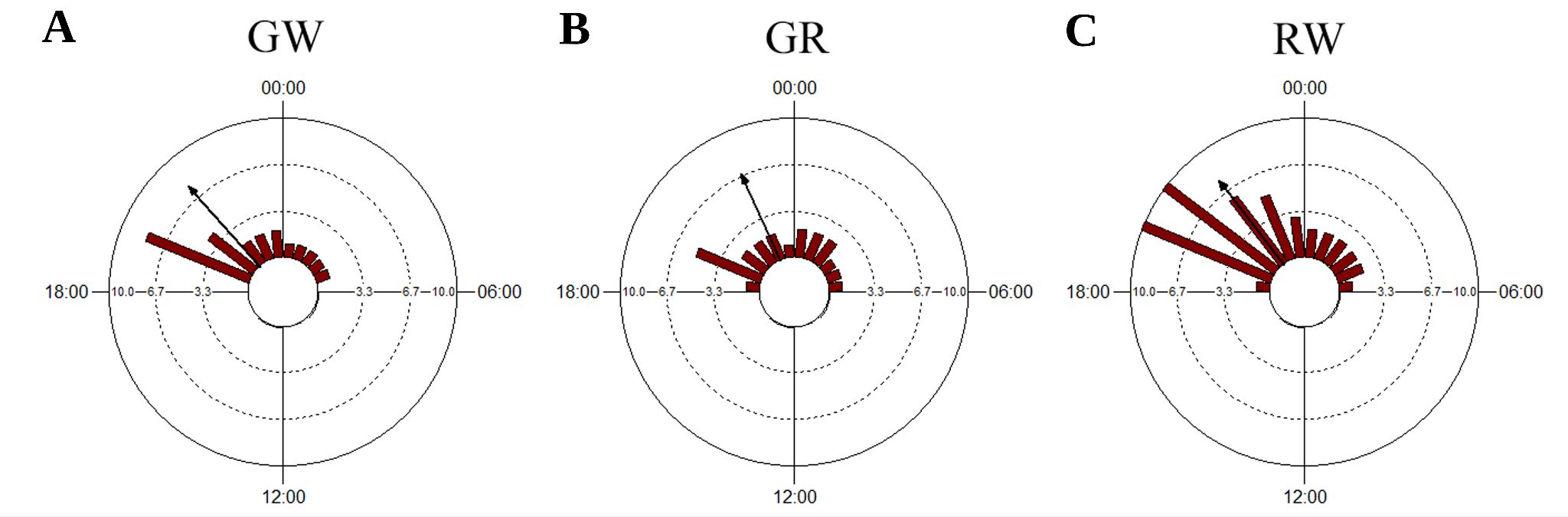


| **Morph** | **GW** | **GR** | **RW** |  | **GW** | **GR** | **RW** |
| --- | --- | --- | --- | --- | --- | --- | --- |
| Mean Vector (µ) | 21:07 (316.834°) | 22:00 (330.036°) | 21:21 (320.378°) | Rayleigh Test (Z) | 56.546 | 66.534 | 108.077 |
| Length of Mean Vector (r) | 0.776 | 0.718 | 0.786 | Rayleigh Test (p) | < 0.0001* | < 0.0001* | < 0.0001* |
| Median | 20:00 (300°) | 22:00 (330°) | 21:00 (315°) | Mardia-Watson-Wheeler Test | | | |
| Concentration | 2.594 | 2.124 | 2.7 |  | W | p-value | Number of observations |
| Circular Variance | 0.224 | 0.282 | 0.214 |  | 3.563 | 0.673 | 398 |
| Circular Standard Deviation | 02:43 (40.847°) | 03:06 (46.621°) | 02:39 (39.776°) |  | GR | GW | RW |
| Standard Error of Mean | 00:16 (4.166°) | 00:16 (4.084°) | 00:11 (2.973°) | GR | ----- | 0.122 | 0.227 |
| 95% Confidence Interval (-/+) for µ | 20:34 (308.667°) | 21:28 (322.029°) | 20:58 (314.55°) | GW | 4.47 | ----- | 0.313 |
|  | 21:40 (325.001°) | 22:32 (338.044°) | 21:44 (326.207°) | RW | 0.621 | 3.321 | ----- |

### Table S1. List of floral and vegetative morphological characters used for the nMDS analysis. From the 33 morphometric characters, 22 characters were converted to ratios in order to avoid pseudoreplication in characters. To avoid multiple correlated variables (denoted by * in the first column), we took ratios of paired variables. Characters which are used in actual analysis are given in the third column and abbreviations for these characters are given in the fourth column.

| **Sr No.** | **Characters** | **Used in the analysis as** | **Abbreviation used in the analysis** |
| --- | --- | --- | --- |
| 1 | Plant height | Plant height | plant_height |
| 2 | Number of leaves | Number of leaves | no_leaves |
| 3* | Lamina length | Ratio (lamina length/lamina width) | lam_ratio |
| 4* | Lamina width |  |  |
| 5* | Inflorescence length | Ratio (inflorescence length/ inflorescence width) | inflo_ratio |
| 6* | Inflorescence width |  |  |
| 7 | Number of open flowers | Number of open flowers | no_open_flo |
| 8* | Peduncle length | Ratio (peduncle length/ peduncle width) | ped_ratio |
| 9* | Peduncle width |  |  |
| 10 | Colour at base of the pseudostem | Colour at base of the pseudostem | col_at_base |
| 11 | Number of coma bracts | Number of coma bracts | no_coma_br |
| 12 | Number of lateral bracts | Number of lateral bracts | no_lat_br |
| 13 | Length of the lateral bracts | Length of the lateral bracts | lat_br_len |
| 14 | Width of the lateral bract | Width of the lateral bract | lat_br_wid |
| 15 | Number of cincinni | Number of cincinni | cincinni |
| 16* | Bracteole length | Ratio (bracteole length/ bracteole width) | brctol_ratio |
| 17* | Bracteole width |  |  |
| 18* | Calyx length | Ratio (calyx length/ calyx width) | cal_ratio |
| 19* | Calyx width |  |  |
| 20* | Corolla tube length | Ratio (corolla tube length/ corolla tube width) | cor_tub_ratio |
| 21* | Corolla tube width |  |  |
| 22* | Corolla lobe length | Ratio (corolla lobe length/ corolla lobe width) | cor_lob_ratio |
| 23* | Corolla lobe width |  |  |
| 24* | Lateral staminode length | Ratio (lateral staminode length/ lateral staminode width) | lat_st_ratio |
| 25* | Lateral staminode width |  |  |
| 26* | Labellum length | Ratio (labellum length/ labellum width) | lab_ratio |
| 27* | Labellum width |  |  |
| 28 | Depth of the notch | Depth of the notch | notch_dep |
| 29* | Anther length | Ratio (anther length/ anther width) | anth_ratio |
| 30* | Anther width |  |  |
| 31 | Style length | Style length | style_len |
| 32* | Nectaries length | Ratio (nectaries length/ nectaries width) | nect_ratio |
| 33* | Nectaries width |  |  |

### Table S2. Results of within-group comparisons for (a) nectar energy (cal), (b) nectar volume and (c) nectar concentration across three morphs of *C. caulina*. Significance was set at 0.05, with significant *p*-values indicated by an asterisk (*).

**a)**

| **Morph** | **Nectar energy (cal)** | | | |  |  |  |  |
| --- | --- | --- | --- | --- | --- | --- | --- | --- |
|  | **Mean** | **SEM** | **Kruskal-Wallis chi squared** | **df** | ***p*-value** | **Comparison between groups (Dunn’s test)** | | |
| GR | 16.7368 | 1.473 | 33.487 | 2 | <0.0001* |  | GR | GW |
| GW | 18.0489 | 2.227 |  |  |  | GW | 0.3178 |  |
| RW | 31.3075 | 2.253 |  |  |  | RW | <0.0001* | <0.0001* |

**b)**

| **Morph** | **Nectar volume (μl)** | |  |  |  |  |  |  |
| --- | --- | --- | --- | --- | --- | --- | --- | --- |
|  | **Mean** | **SEM** | **Kruskal-Wallis chi squared** | **df** | ***p*-value** | **Comparison between groups (Dunn’s test)** | | |
| GR | 11.8296 | 0.866 | 32.619 | 2 | <0.0001* |  | GR | GW |
| GW | 13.2484 | 1.375 |  |  |  | GW | 0.1936 |  |
| RW | 21.0043 | 1.38 |  |  |  | RW | <0.0001* | <0.0001* |

**c)**

| **Morph** | **Nectar concentration (% sucrose)** | |  |  |  |  |  |  |
| --- | --- | --- | --- | --- | --- | --- | --- | --- |
|  | **Mean** | **SEM** | **Kruskal-Wallis chi squared** | **df** | ***p*-value** | **Comparison between groups (Dunn’s test)** | | |
| GR | 30.7053 | 0.654 | 10.13 | 2 | 0.006315* |  | GR | GW |
| GW | 28.6438 | 0.967 |  |  |  | GW | 0.0498* |  |
| RW | 32.5056 | 0.504 |  |  |  | RW | 0.0521 | 0.0007* |

### Table S3. Results of within-group comparisons for peak pollinator visitation rates (# visits per flower per hour) for all three morphs of *C. caulina*. Significance was set at 0.05, with significant *p*-values indicated by an asterisk (*).

| **Kruskal-Wallis chi squared** | **df** | ***p*-value** | **Comparison between groups (Dunn’s test)** | | |
| --- | --- | --- | --- | --- | --- |
| 6.52 | 2 | 0.03839* |  | GR | GW |
|  |  |  | GW | 0.3377 |  |
|  |  |  | RW | 0.0066* | 0.0302* |

### Table S4. Results of chi-square test for success rates of self- and cross-compatibility treatments conducted for all three morphs. Significance was set at 0.05, with significant *p*-values indicated by an asterisk (*).

| **Treatment** | **Effect** | **Chi-square value** | **df** | ***p*-value** |
| --- | --- | --- | --- | --- |
| All three | Treatments | 29.278 | 1 | < 0.0001* |
| Self-pollination | Female morph | 2.8681 | 2 | 0.2383 |
| Intra-morph cross-pollination | Male/ female morph | 5.5019 | 2 | 0.06387 |
| Inter-morph cross-pollination | Pair a: GW and GR morphs | 1.5771 | 1 | 0.2092 |
|  | Pair b: GW and RW morphs | 2.2711 | 1 | 0.1318 |
|  | Pair c: GR and RW morphs | 0.22996 | 1 | 0.6316 |

### Table S5. Success rate, mean and standard error (s.e.) values for seed-count for cross- and self-compatibility treatments conducted for all three morphs.

| **Treatment** | **Male** | **Female** | **Success rate (%)** | **Mean** | **SEM** |
| --- | --- | --- | --- | --- | --- |
| Self-pollination | Self | GR | 0 | 0 | 0 |
|  | Self | GW | 6.67 | 14.5 | 6.5 |
|  | Self | RW | 9.68 | 13.334 | 4.372 |
| Intra-morph cross pollination | GR | GR | 46.67 | 20.714 | 2.825 |
|  | GW | GW | 18.75 | 10.167 | 2.713 |
|  | RW | RW | 27.03 | 15.7 | 2.712 |
| Inter-morph cross pollination | GR | GW | 43.33 | 20.846 | 2.557 |
|  | GR | RW | 37.84 | 20.429 | 3.295 |
|  | GW | GR | 25 | 15 | 2.872 |
|  | GW | RW | 24.24 | 12.125 | 2.553 |
|  | RW | GR | 46.67 | 21.5 | 3.122 |
|  | RW | GW | 44.44 | 17.813 | 2.771 |

### Table S6. Results of between groups comparisons for seed-count for (a) self-pollination, (b) intra-morph cross-pollination and (c) inter-morph cross-pollination treatments for all three morphs. Significance was set at 0.05, with significant *p*-values indicated by an asterisk (*).

**a)** Kruskal-Wallis chi-squared = 5.3534, df = 2, *p*-value = 0.06879

| **Female morphs** |  | GR | GW |
| --- | --- | --- | --- |
|  | GW | 0.0419* |  |
|  | RW | 0.0158* | 0.4238 |

**b)** Kruskal-Wallis chi-squared = 5.2499, df = 2, *p*-value = 0.07244

| **Intra-morph cross** |  | GR x GR | GW x GW |
| --- | --- | --- | --- |
|  | GW x GW | 0.0115* |  |
|  | RW x RW | 0.1419 | 0.0987 |

**c)**

| **Inter-morph pairs** | | **Kruskal-Wallis chi-squared** | ***p*-value** |
| --- | --- | --- | --- |
| a | GR x GW | 1.5177 | 0.218 |
| b | RW x GW | 1.3614 | 0.2433 |
| c | RW x GR | 0.03389 | 0.8539 |

#

# **Table S7:** Results of chi-square statistics tests for model fits for all four SEM models.

| **Model** | **Population** | **GR Morph** | **GW morph** | **RW morph** |
| --- | --- | --- | --- | --- |
| Chi-square statistics | 0.052 | 0.037 | 0.006 | 0.664 |
| p-value (Chi-square) | 0.82 | 0.847 | 0.94 | 0.415 |
| CFI value | 1 | 1 | 1 | 1 |
| TLI value | 1.332 | 7.138 | -0.65 | 1.494 |

# 
